# A fully automated image analysis method to quantify lung fibrosis in the bleomycin-induced rat model

**DOI:** 10.1371/journal.pone.0193057

**Published:** 2018-03-16

**Authors:** Shanon Seger, Manuel Stritt, Enrico Vezzali, Oliver Nayler, Patrick Hess, Peter M. A. Groenen, Anna K. Stalder

**Affiliations:** 1 Drug Discovery Biology, Idorsia Pharmaceuticals Ltd. Hegenheimermattweg, CH, Allschwil, Switzerland; 2 Drug Discovery Pharmacology, Idorsia Pharmaceuticals Ltd. Hegenheimermattweg, CH, Allschwil, Switzerland; Pennsylvania State Hershey College of Medicine, UNITED STATES

## Abstract

Intratracheal administration of bleomycin induces fibrosis in the lung, which is mainly assessed by histopathological grading that is subjective. Current literature highlights the need of reproducible and quantitative pulmonary fibrosis analysis. If some quantitative studies looked at fibrosis parameters separately, none of them quantitatively assessed both aspects: lung tissue remodeling and collagenization. To ensure reliable quantification, support vector machine learning was used on digitalized images to design a fully automated method that analyzes two important aspects of lung fibrosis: (i) areas having substantial tissue remodeling with appearance of dense fibrotic masses and (ii) collagen deposition. Fibrotic masses were identified on low magnification images and collagen detection was performed at high magnification. To insure a fully automated application the tissue classifier was trained on several independent studies that were performed over a period of four years. The detection method generates two different values that can be used to quantify lung fibrosis development: (i) percent area of fibrotic masses and (ii) percent of alveolar collagen. These two parameters were validated using independent studies from bleomycin- and saline-treated animals. A significant change of these lung fibrosis quantification parameters- increased amount of fibrotic masses and increased collagen deposition- were observed upon intratracheal administration of bleomycin and subsequent significant beneficial treatments effects were observed with BIBF-1120 and pirfenidone.

## Introduction

Idiopathic Pulmonary Fibrosis (IPF) is a chronic and progressive human lung disease with unclear etiology and poor prognosis and is one of the most severe form of all interstitial pneumonias.[1] IPF is characterized by endothelial and epithelial damages, inflammation with neutrophils, macrophages and lymphocytes, followed by abnormal fibroblasts proliferation and collagen deposition in lung parenchyma. This disease results in increasing loss of lung function and ultimately to death. Although two anti-fibrotic compounds, pirfenidone (Esbriet®) and BIBF-1120/nintedanib (OFEV®) reduced lung function decline in IPF patients and have recently been approved for the treatment of IPF they do not offer a cure.[2, 3] Intra-tracheal (IT) instillation of bleomycin in rat is a widely used experimental model to study mechanisms of pulmonary fibrosis and to evaluate novel treatment modalities.[4] Instillation of bleomycin induces a multiphasic process that is initiated by an acute and transient inflammatory response followed by a persisting fibrotic response.[5]

Current literature on pulmonary fibrosis in rodents and in human clinical samples highlights the necessity to have an accurate assessment of lung fibrosis severity. The current gold standard to assess lung fibrosis is histopathological analysis. Analysis is performed manually by pathologists using Masson's trichrome-stained paraffin sections that are observed under a microscope. Ashcroft et al developed the first reliable semi-quantitative scoring system based on microscopy hyper fields to assess the severity of lung fibrosis in human samples.^[^^6^^]^ The Ashcroft numerical scale is composed of 9 severity degrees from 0 (normal lung) to 8 (total fibrous obliteration) based on the assessment of multiple parameters in several microscopic high power fields. Fibrosis is a multi-phasic process starting with thickening of alveolar walls, followed by accumulation of damage to lung architecture leading finally to fibrotic masses and emphysema.[6] Although Ashcroft's scale was originally developed to assess human tissue it has been widely used in bleomycin-induced pulmonary fibrosis in animal models, but is compromised by inter-observer and intra-observer variability. To allow for a translatable read-out for animal model research, Hübner modified the Ashcroft’s scale for bleomycin induced fibrosis in rats.[7] This new scale maintains the same granularity as the Ashcroft scale but better describes each grade with separate observations focusing on lung structures and alveolar septa. Besides having a reliable scoring system of lung fibrosis, analysis of separated lung fibrosis features could be standardized by automated quantification.

To date, most of available automated quantifications of lung fibrosis are based on *in vivo* micro-computed tomography (μCT) image analysis. [8–10] Ex-vivo digital imaging is available since more than 30 years, however initial poor image quality has limited its wide spread application. Subsequent innovation in acquisition devices and whole slide imaging development allowed high resolution bright field images suitable for digital pathology image analysis. [11, 12] The development of whole slide scanners allows researcher and pathologist to characterize and quantify fibrosis in the entire lung. The large size of these digitalized images and high animal numbers needed for studies highlight the need for automated methods to study lung fibrosis. In pre-clinical research, studying pharmacological effects of novel compounds requires reproducible readouts over multiple studies. An automated process, which is independent from human subjectivity over time to study individual aspects of fibrosis features, would therefore be an important asset. Methods currently utilized to investigate lung fibrosis have limitations. No current automated methods using histological staining investigate the multi-parametric aspects of lung fibrosis (alveolar collagen deposition and fibrotic masses). Carai et al. and Egger et al. assess lung collagenization parameter using a color content/treshold based analysis [13, 14] whereas Kumar et al. and Gilhodes et al. focus their analysis on morphological changes in the lung [15, 16].

Here, we describe the development of a standardized and fully automated histo-morphometric image analysis methodology to quantify lung fibrosis in bleomycin-treated rats. The method is based on the two main parameters described in the modified Ashcroft numerical scale: thickening of alveolar walls by collagen deposition and fibrotic masses. The separation of both markers provides further mechanistic information when studying compound effects.

In contrast to previous methods where only colored area detection for fibrosis processes or tissue markers were used,[12] the procedure presented here uses support vector machine learning (SVM) to train the computer to detect not only colored areas but also tissue structure. SVM learning is a powerful tool in image analysis already used in digital pathology to support histological diagnosis in other disease areas such as neuropathology.[17]

## Materials and method

### Animals

Normotensive male Wistar rats were obtained from Harlan Laboratories (Horst, the Netherlands) and male Sprague Dawley were obtained from Charles River laboratories (Sulzfeld, Germany. Rats were group housed with appropriate environmental enrichment (shelter, tunnel, and wooden blocks). All animals were maintained under identical conditions and had free access to drinking water and normal pelleted food. Animals were housed in climate-controlled conditions (18–22°C and 40–60% humidity) with a 12-h light/dark. Well-being of animals was monitored during the day by technical assistants. Animals were checked on body weight loss, abnormal breathing, pilo-erection, grooming and locomotion as criteria of distress following our internal animal welfare policy and guideline on humane endpoints.

All of the experimental procedures were conducted in accordance with the Swiss animal welfare ordinance and Actelion Animal Welfare policy on the use of experimental animals. The study was approved by the Basellandschaft Cantonal Veterinary Home Office (license no. 371).

Animals were acclimatized for at least 7 days prior to experiment. Bleomycin (1.5–1.7 and 2 mg/kg, Baxter, Switzerland) or saline instillation was done at Day 0. Treatments: pirfenidone (Esbriet®) 0.5% in powder food or control, BIBF-1120/nintedanib (OFEV®) 50mg/kg p.o. or vehicle (gelatin 7.5%). Sacrifices were performed at day 14 and 28. The animals were anesthetized with Isoflurane (5%) and exsanguinated and lungs left lobe were collected for histology (Table 1).

**Table 1:** Animal studies overview.

	Study 1	Study 2	Study 3	Study 4	study 5	Study 6
Staining periode	2012	2013	early 2014	early 2014	end 2014	2016
**Rat strain**	Sprague Dawley	Wistar	Wistar	Wistar	Wistar	Wistar
**bleomycin dose**	1.7mg/kg	2mg/kg	2mg/kg	2mg/kg	1.5mg/kg	1.5mg/kg
**BIBF-1120**	50mg	50mg				50mg
**pirfenidone**		0.50%		0.50%		0.50%
**Necropsy**	D28	D14	D14-28	D28	D28	D14

Six independent studies performed in-house over 4 years were used for method establishment and validation.

### Histology

Lung left lobes were instilled with 4ml of 4% formaldehyde and fixed under pressure for 48h at room temperature, embedded in paraffin blocks and sectioned at 2μm. Masson’s trichrome staining was performed using an automated tissue stainer TST44 (Medite Gmbh, Burgdorf, Germany). Staining protocol: Iron Hematoxylin for 5 minutes, tap water for 5 minutes, rinse with distilled water, Ponceau-Acid fuchsine-Azophloxin for 7 minutes, rinse 2 times with 1% CH3COOH, differentiate in phosphotungstic acid-Orange G for 10 minutes, rinse 2 times with 1% CH3COOH, counterstain with waterblue 5 minutes, rinse 1 time with 1% CH3COOH, place in 1% CH3COOH for 2 minutes, wash 2x in EtOH, dehydrate before mounting the slides. dx.doi.org/10.17504/protocols.io.mdhc236.

### Image acquisition

Histology slides were digitalized using Nanozoomer 2.0HT whole slides scanner (Hamamatsu Photonics, Japan). Scans were performed using the fully automated mode and 40x objective. Images were uploaded in Orbit Image Analysis software (Actelion Pharmaceuticals Ltd, Allschwil, Switzerland).[18] Orbit Image Analysis software requires a minimum of 8GB of RAM to insure proper visualization of images, and computation.

### Digital image set

The method development training set was composed of 20 images of independent slides covering observed staining variation, slides were collected from study 1 to 5. To establish and validate the new automated image analysis methodology, we used slide images from digitized whole mount glass slides (n = 190) from 6 independent studies performed over 4 years (Table 1).

### Image analysis

Orbit Image Analysis software (Actelion Pharmaceuticals Ltd)[18] has been developed for whole slide image analysis. An open-source version has been released for common use under the GPL license. Its main focus is to perform machine learning based classification and segmentation making use of multi resolution image pyramids. An SVM based classifier is used for pixel classification based on multivariate intensity and structural input parameters.

For each pixel a certain region defined by the structure size is taken into account to compute features which are used as input for the SVM. The features include structural descriptors such as edge factor or the variance and intensity features such as mean, min and max intensity per channel. For example, the edge factor is computed as

$$\sqrt{\frac{1}{|W|-1}\sum_{p^{\prime }\;\epsilon \;W}^{}{\left(p^{\prime }-p\right)}^{2}}$$

where W is the set of all pixels within the surrounding area of pixel p.

A training step was done by manually drawing several representative annotations per tissue class (e.g. collagen, normal tissue,). For each pixel within the union of all training regions the features are computed and define the training set which was used to train the SVM.

The classification step then works automatically: the features for each pixel with the valid ROI are computed and the SVM outputs the corresponding tissue class.

This procedure can be applied at different magnifications of the image. A so-called exclusion map was computed to define the region of interest (ROI). This classification map classifies pixels on lower magnification (around 1 megapixel) using a large structure size which allows to discriminate a larger context.

Within the valid ROI a fine grained classification model was applied on the full resolution image (around 6000 megapixels). Since the image is very big and would not fit into memory, the whole image is divided into tiles of 512x512 pixels. Each tile is analyzed in a map-reduce manner using our in-house grid infrastructure: the map step computes the tissue class ratios per tile, the reduce step combines the tile results.

### Statistics

Results are presented as mean +/- SEM using Prism 6 (GraphPad Software, Inc.). P values are calculated with an unpaired T test and using a non-parametric test (Mann-Whitney test).

## Results

### Method development

**Fig 1:**
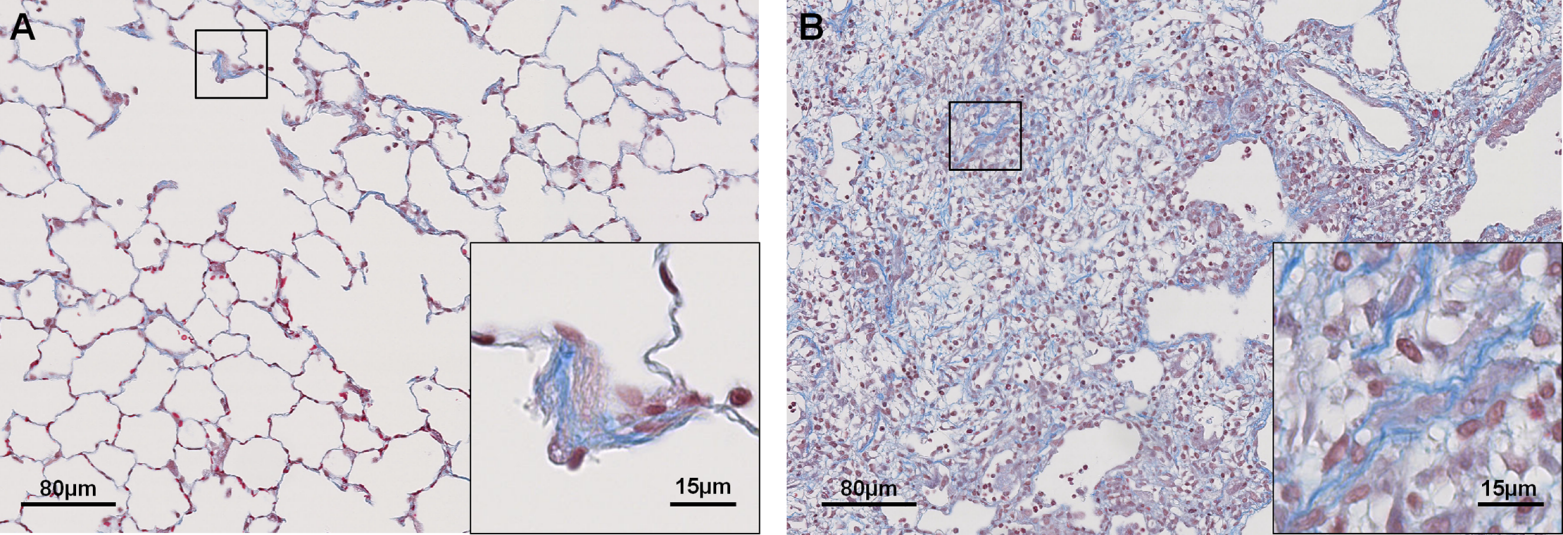
Fibrosis features used for morphometric detection. Digitalized slides of Masson’s tri-chrome stained lung paraffin sections. Collagen is stained in blue and cell nuclei in red. **A**: alveolar thickening by collagen deposition. **B**: fibrotic masses composed of collagen, fibroblasts, and other components.

Identification of the two main criteria that describe lung fibrosis in bleomycin instilled rats is based on the Hübner numerical scale to study fibrosis in rodent. In early stages lung fibrosis is characterized by collagen deposition in alveolar walls and later on with disease progression fibrotic masses are found in addition to alveolar collagen. These two criteria are easily visualized on a Masson’s tri-chrome histological stain (Fig 1).

Pictures extracted from five independent in-house studies that spanned over four years, covering a wide range of staining variation in color and intensities (S1 Fig), were used to train a robust tissue detection classifier combining two detection models to output two lung fibrosis features: fibrotic masses and alveolar collagen.

**Fig 2:**
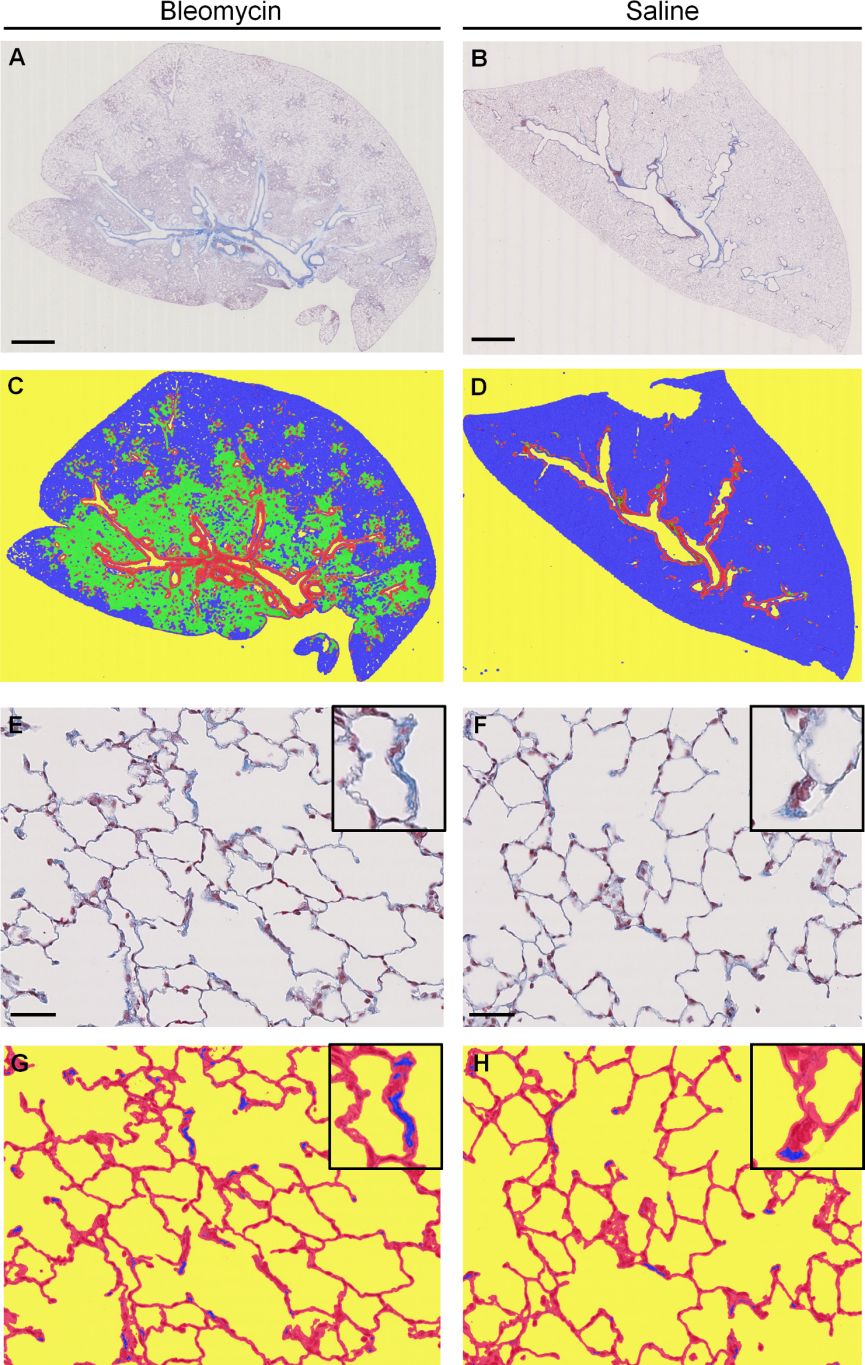
Fibrosis feature detection. Masson’s trichrome stained lung sections from study 3, Bleomycin (A, C, E, F) and Saline (B, D, F, H) animals, **A and B** at low magnification, scale bar 2.5mm **E and F** alveolar structure at high magnification, scale bar 50μm. Upper right square: zoom in one alveola. **C and D**: low scale detection model; false colour image indicates areas of dense fibrosis (green), alveolar tissue (blue), bronchi (red), background (yellow). **G and H:** high magnification detection; false colour image indicates collagen (blue), lung tissue (red), background (yellow). Upper right square: zoom in one alveola collagen detection.

The first detection model discriminates 4 classes on the low magnification image: background (non-tissue), fibrotic masses, alveolar tissue and bronchi (plus its surrounding collagen). This model computes the percentage of fibrotic masses per lung. This step additionally defines the region of interest (ROI) used for collagen detection. In this case the ROI is the alveolar tissue excluding the bronchi and surrounding collagen class (Fig 2A–2D).

The second model focuses on quantification of collagen deposition. *De novo* collagen is detected by tissue classification on high magnification images (Fig 2E–2H) inside alveolar tissue. As the first detection model defines the region of interest for collagen detection, total lung collagen can also be produced as additional read out.

Annotations for every tissue class were manually drawn by the investigator on pictures composing the training set. SVM learning was applied on this subset of images. 40 000 randomly picked pixel (over every class and picture) were used for computation and classification. The model was evaluated by a 10-fold cross-validation. For this, 9/10 of the randomized training samples was used to train the model. The remaining 1/10 of the data was used to test the accuracy. This procedure was repeated in a round-robin way 10 times to test all the data, but always while training the model on unseen data. Annotations and training steps were repeated until a proper tissue classification over the whole study set was achieved by a manual assessment of classified pictures (verified by a pathologist). The average accuracy over all folds was 95.47% insuring that the model is able to classify the tissue structures exactly as defined by the investigator.

### Inter study assay stability

To ensure an independent and automated use of the method in all studies within the staining range, image analysis stability was assessed on studies used for method development and an additional independent study was included (Table 1, study 6). Two time points were studied and for each time point, three independent studies were analyzed. A visual quality check was performed to exclude collapsed lungs from the quantification (non-instilled lungs). All studies were run as a batch for fully automated quantification and the two parameters were read out as: fibrotic masses and alveolar collagen.

**Fig 3:**
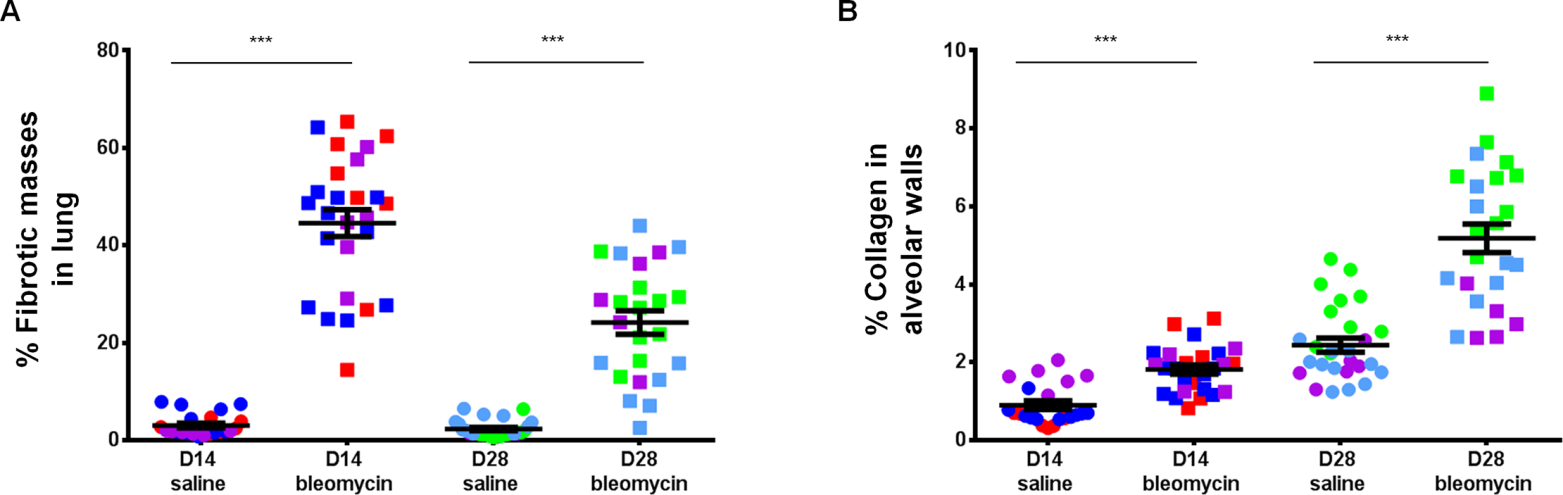
Inter study reproducibility of the quantification method. Automated image analysis was applied to five independent studies, performed over 2 years (3 independent studies for each time point: D14 and D28). **A**: percentage of fibrotic masses per lung (excluding bronchi for normalization), **B**: percentage of alveolar collagen deposition. Mean with +/- SEM, Mann-Whitney statistical test. Each dot represents an individual animal and color represents independent studies. Study 2: red, study 3: purple, study 4: dark blue, study 5: green, and study 6: light blue.

A clear induction of morphological changes was seen with both parameters in bleomycin treated groups at both time points: Day 14 and Day 28 (Fig 3A and 3B). Saline-treated control animals did not show fibrotic masses (mean: D14 = 3.05, D28 = 2.293). Bleomycin-treated animals showed a statistically significant increase in percent fibrotic mass per lung at Day 14 (mean = 44.57, P<0.0001). At Day 28 the amount of fibrotic masses remained significantly increased compared to saline-treated animals (mean = 24.16, P<0.0001), although it was less than that observed at Day 14 (Fig 3A). These findings were consistent across three independent studies as evident by the overlap of animals from independent experiments.

Similar consistency was observed with collagen measurements over independent studies, confirming the assay stability for collagen detection independent from disease duration (Fig 3B). Bleomycin instillation induced a statistical collagen deposition in alveolar parenchyma at both time points, D14 and D28 (P<0.0001 for both; mean D14: saline = 0.90, Bleomycin = 1.81, D28: saline = 2.44, Bleomycin = 5.18). A statistical increase of collagen in alveolar walls (P<0.0001) between Day 14 and Day 28 was observed in control animals from saline groups. A statistical increase of collagen over time was also observed in bleomycin treated animals (P<0.0001). When comparing time points, a tight grouping of animals from saline and bleomycin groups was observed at Day 14 compared to a less tight spread at Day 28. This difference in scattering between time points was only observed for the collagen measurement and not in fibrotic masses. Both parameters were stable over studies and independent of bleomycin disease duration.

### A sensitive method to describe anti-fibrotic compound effects

**Fig 4:**
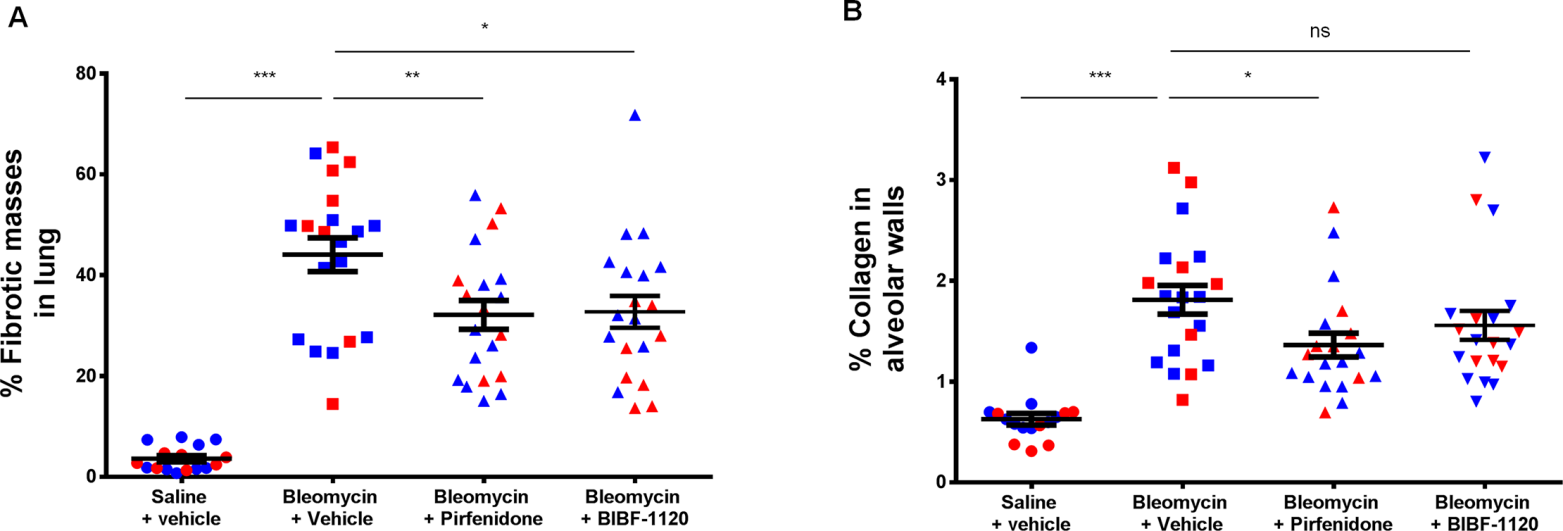
Quantitative analysis of anti-fibrotic compound effects. Lungs from Wistar rats treated with bleomycin in combination with pirfenidone (0,50%) and BIBF-1120 (50mg/kg) were collected at Day 14 and a Masson’s trichrome staining was performed for fibrosis parameter assessment using automated image analysis. **A**: percentage of fibrotic masses, **B**: percentage of alveolar collagen. Mean with +/- SEM, Mann-Whitney statistical test. Each dot represents an individual animal and color represents independent studies. Study 2 in red, and study 6 in blue.

To assess a possible application of the method to evaluate the effect of pharmacologic compounds on fibrosis, two anti-fibrotic drugs approved for lung fibrosis treatments were used as positive controls: BiBF-1120 and pirfenidone. One section per animal and 18–20 animals per group was analyzed using the fully automated assay. An increase in fibrotic mass and collagen was observed in bleomycin treated animals compared to saline (P<0.0001) (Fig 4A and 4B). Both anti-fibrotic compounds significantly reduced fibrotic masses in lungs when compared to untreated animals (Fig 4A). When analyzing alveolar collagen, only pirfenidone exhibited a beneficial effect (P = 0.0167). (Fig 4B). Similar pattern was observed in Sprague Dawley rats (S2 Fig).

## Discussion

Bleomycin experimentally induces lung injury and fibrosis in animal models. Gene set enrichment analysis showed direct relevant molecular changes induced by bleomycin when compared to human IPF active disease signature.[19] For the method described here to quantify lung fibrosis, an intra-tracheal fibrosis rat model was used but a similar strategy could be applied to other species or disease induction methods. This model is widely used to study lung fibrosis and to evaluate novel treatments.[4] The histology-based scoring for the assessment of lung fibrosis mechanism and progression in rodents takes into consideration two important parameters of fibrosis: alveolar septa thickening and lung structure changes. These are assessed together with other parameters of fibrosis into a multi-parameter score. [7]

Using SVM learning, a methodology for automatic quantification of these lung fibrosis parameters separately was developed to better quantify pathologic changes during fibrosis and to evaluate the anti-fibrotic effect of drugs. Using low magnification pictures, the computer uses the trained model to identify the tissue and differentiate lung tissue structures: airways (bronchi and bronchiole), blood, fibrotic masses and alveoli. One major advantage of SVM is the recognition and exclusion of structures which pathologist would spontaneously ignore as belonging to pre-existing normal anatomy under physiological conditions. Collagen from airways and their surrounding are excluded from the quantification because bronchi representation in sections from different animal is variable and therefore normal collagen could influence the investigated change in collagen due to the induction of fibrosis and the consequent treatment effect of anti-fibrotic compounds. In contrary to the image analysis described by Gilhodes et al, this method automatically exclude bronchi and vessel and do not require a manual input by the user apart from the exclusion of non-inflated lungs.[16]

Histological staining variability is a well-known issue and can be caused by different parameters such as slide preparation, tissue thickness and staining (Pathology vision 2015, Boston, USA). In-house staining was performed using an automated stainer to minimize possible variations.

Numerous tools exist to study tissue fibrosis, and most of them focuses on collagen content of the tissue using threshold based algorithm provided by Image J (NIH) (with grayscale or color threshold), tissuemorph DP (Visiopharm, Horsholm, Denmark), or Histolab (microvision Instruments, Evry, France).[14, 20] Some companies, have focused their analysis on fibrotic masses using tissue density (Biocellvia, Marseille, France).[16] In contrary to other image analysis based methods such as color content analysis or tissue density analysis the method described here combines several analysis parameters used at different image resolution to study both fibrosis parameters. These include: single pixels mean, maximum, and minimum intensity per color; applying similar computation to surrounding pixels forming a structure; variance and edge factor within a defined structure size. All of these parameters are used by the computer during the training phase in order to classify the anatomical structures within a tissue class defined by the rater.[18] In this study, machine learning was applied to a wide training set covering staining and biological variability to obtain a robust assay to study tissue structural changes observed in the fibrotic process of lung within an acceptable range of staining. The detection method was reproducible and showed consistency in independent studies even when used automatically (without further training of the model). The method can be used independent of the disease stage as shown here at Day 14 and 28 after bleomycin treatment. The production of reproducible and standardized intra- and inter-study data, allows for a direct comparison between animal study results and compound effects. Studies using the same experimental design could also be pooled.

A tight grouping of saline animals and a scattering of animals from bleomycin group was observed for both fibrosis parameters. This observation was reproducible in all studies. Bleomycin variability in the induction of inflammation and collagen deposition is known, as well as the non-homogeneous distribution of the intra-tracheal instilled bleomycin solution leading to the observed scattering of parameters in bleomycin animal group. [21]

BiBF-1120 and pirfenidone are two anti-fibrotic drugs that recently obtained regulatory approval for the treatment of lung fibrosis in man. Their anti-fibrotic effects are routinely studied in rat intra-tracheal bleomycin induced lung fibrosis. Using the automated method described here to evaluate fibrotic mass and collagen deposition, both drugs showed an anti-fibrotic effect by reducing fibrotic masses, but only pirfenidone was able to significantly reduce alveolar collagen. Total lung collagen could also be analyzed using this automated image analysis method with exclusion of bronchi surrounding constitutive collagen and therefore exhibiting superior results compared to other biochemical assays such as sircol kit and hydroxyproline. Indeed, our data show that evaluating separate fibrosis parameters when studying different anti-fibrotic compound via purely histological read-outs may unmask different mechanisms of action for these compounds.

Fibrotic masses are composed of fibroblasts, myofibroblasts, collagen fibers and inflammatory cells such as T cells and B cells. [19, 22, 23] Pirfenidone positive effects observed on fibrotic masses are probably linked to the reduction of inflammation and the decrease of fibroblast proliferation observed in previous studies using molecular read-outs. Pirfenidone is known to have an anti-inflammatory effect by down regulating interleukins and an anti-fibrotic effect by reduction of fibroblast proliferation via lung basic-fibroblast growth factor (bFGF) and transforming growth factor (TGF)-beta1 levels.[24] BiBF-1120 is a triple intra-cellular tyrosine kinase inhibitor. It targets Fibroblast growth factor receptor (FGFR), vascular endothelial growth factor receptor (VEGFR) and platelet derived growth factor receptor (PDGFR) reducing and the inflammatory score of lungs from bleomycin treated rodents models.[25] A positive effect by pirfenidone on alveolar collagen is in line with previous studies. Pirfenidone reduced collagen content of mouse lungs after bleomycin administration using sircol collagen assay.[26] Pirfenidone's beneficial effect on collagen deposition is also described for other fibrotic diseases where it reduces collagen type I mRNA levels in fibrotic livers and hearts.[27, 28] BiBF-1120 seems only to have a significant effect on collagen when used in a preventive context and not therapeutically.[25]

## Conclusion

For the first time, an histological image analysis assay analyzing both aspects of fibrosis, structural changes (fibrotic masses accumulation) and molecular changes (collagen deposition within the alveolar septae) is described. Analysis was performed in a fully automated manner and applicable to all Masson’s trichrome stained rat lungs within a specified staining range. This assay does not need further input from the user and can be used as a module (dx.doi.org/10.17504/protocols.io.neudbew) in ORBIT Image Analysis open source software (http://www.orbit.bio, May 17^th^ 2017). This histological assay showed stability over time, reproducibility and reliability of results. The separation of 2 fibrosis parameter increases sensitivity and granularity when compared to composit scores and allows to study compound effects.

## Supporting information

S1 Fig Representative picture of Masson’s trichrome staining variation over 4 years of studies. Lungs from bleomycin treated animals. Pictures from the top raw represents strong staining intensities with different colour spectrum **A** reddish, **B** orange, and **C** bluish. Second raw represents the gradual shift of staining from blue **D** to greye **F**. Scale bare 40μm.
(TIF)

S2 Fig Quantitative analysis of anti-fibrotic compound effects on a second rat strain (Sprague Dawley). Lungs from Sprague Dawley rats treated with bleomycin in combination with BIBF-1120 (50mg/kg) were collected at Day 28 and a Masson’s trichrome staining was performed for fibrosis parameter assessment using automated image analysis. **A**: percentage of fibrotic masses, **B**: percentage of alveolar collagen. Mean with +/- SEM, Mann-Whitney statistical test.
(TIF)
