# Supplementary figures and images for: A fully automated image analysis method to quantify lung fibrosis in the bleomycin-induced rat model
Source: PLoS One. 2018 Mar 16;13(3):e0193057. doi: 10.1371/journal.pone.0193057 (PMC5856260; doi:10.1371/journal.pone.0193057)

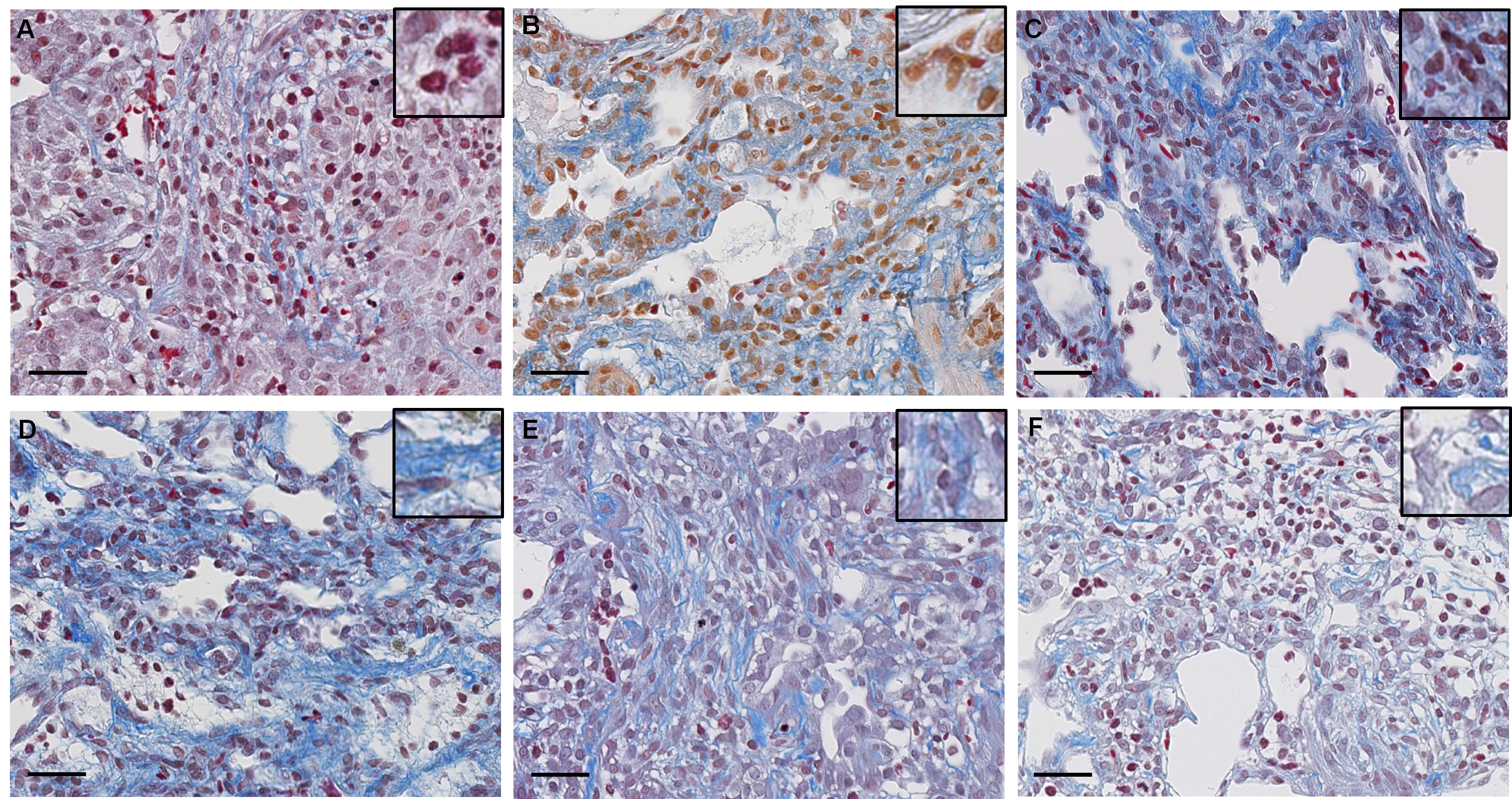

Supplement: S1 Fig — Lungs from bleomycin treated animals. Pictures from the top raw represents strong staining intensities with different colour spectrum A reddish, B orange, and C bluish. Second raw represents the gradual shift of staining from blue D to greye F. Scale bare 40μm. (TIF) [file pone.0193057.s001.tif]

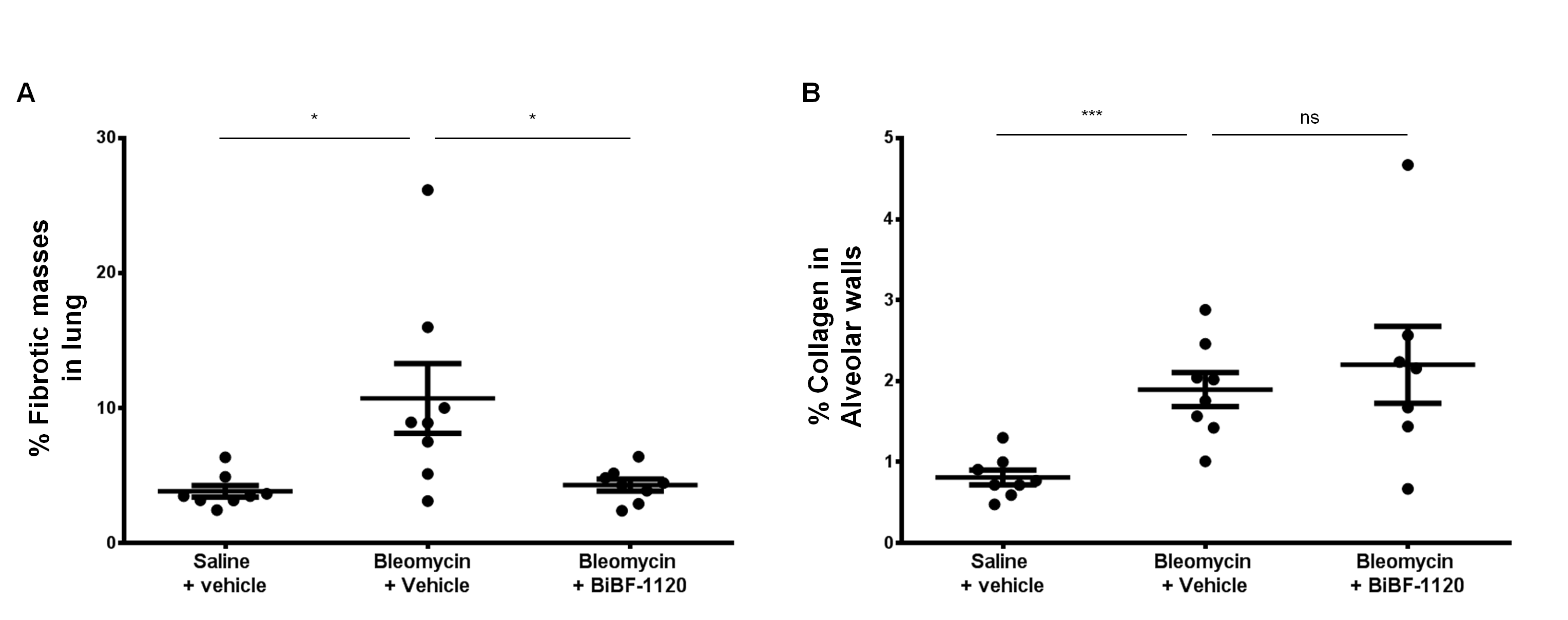

Supplement: S2 Fig — Lungs from Sprague Dawley rats treated with bleomycin in combination with BIBF-1120 (50mg/kg) were collected at Day 28 and a Masson’s trichrome staining was performed for fibrosis parameter assessment using automated image analysis. A: percentage of fibrotic masses, B: percentage of alveolar collagen. Mean with +/- SEM, Mann-Whitney statistical test. (TIF) [file pone.0193057.s002.tif]
